# Comparative Genomics Analysis of *Lactobacillus ruminis* from Different Niches

**DOI:** 10.3390/genes11010070

**Published:** 2020-01-08

**Authors:** Shuo Wang, Bo Yang, R. Paul Ross, Catherine Stanton, Jianxin Zhao, Hao Zhang, Wei Chen

**Affiliations:** 1 State Key Laboratory of Food Science and Technology, Jiangnan University, Wuxi 214122, China; ws87978797@163.com (S.W.); zhaojianxin@jiangnan.edu.cn (J.Z.); zhanghao61@jiangnan.edu.cn (H.Z.); chenwei66@jiangnan.edu.cn (W.C.); 2 School of Food Science and Technology, Jiangnan University, Wuxi 214122, China; 3 International Joint Research Center for Probiotics & Gut Health, Jiangnan University, Wuxi 214122, China; p.ross@ucc.ie (R.P.R.); catherine.stanton@teagasc.ie (C.S.); 4 APC Microbiome Ireland, University College Cork, Cork, T12K8AF Cork, Ireland; 5 Teagasc Food Research Centre, Moorepark, Fermoy, P61C996 Co. Cork, Ireland; 6 National Engineering Research Center for Functional Food, Jiangnan University, Wuxi 214122, China; 7 Wuxi Translational Medicine Research Center and Jiangsu Translational Medicine Research Institute Wuxi Branch, Wuxi Branch, Wuxi 214122, China; 8 Beijing Innovation Center of Food Nutrition and Human Health, Beijing Technology and Business University (BTBU), Beijing 102488, China

**Keywords:** *Lactobacillus ruminis*, phylogenetic relationship, bacteriocins, carbohydrate utilization, CRISPR/Cas, prophage

## Abstract

*Lactobacillus ruminis* is a commensal motile lactic acid bacterium living in the intestinal tract of humans and animals. Although a few genomes of *L. ruminis* were published, most of them were animal derived. To explore the genetic diversity and potential niche-specific adaptation changes of *L. ruminis*, in the current work, draft genomes of 81 *L. ruminis* strains isolated from human, bovine, piglet, and other animals were sequenced, and comparative genomic analysis was performed. The genome size and GC content of *L. ruminis* on average were 2.16 Mb and 43.65%, respectively. Both the origin and the sampling distance of these strains had a great influence on the phylogenetic relationship. For carbohydrate utilization, the human-derived *L. ruminis* strains had a higher consistency in the utilization of carbon source compared to the animal-derived strains. *L. ruminis* mainly increased the competitiveness of niches by producing class II bacteriocins. The type of clustered regularly interspaced short palindromic repeats /CRISPR-associated (CRISPR/Cas) system presented in *L. ruminis* was mainly subtype IIA. The diversity of CRISPR/Cas locus depended on the high denaturation of spacer number and sequence, although cas1 protein was relatively conservative. The genetic differences in those newly sequenced *L. ruminis* strains highlighted the gene gains and losses attributed to niche adaptations.

## 1. Introduction

*Lactobacillus ruminis* is a lactic acid bacterium which is phylogenetically close to *Lactobacillus salivarius* [1], and is strictly anaerobic with low GC content [2]. *L. ruminis* was firstly isolated from human feces in 1960 and originally identified as *Catenabacterium catenaforme* [3], which was subsequently isolated from bovine rumen [4]. *L. ruminis* can trigger certain protective responses in humans and animals when given as a probiotic or symbiotic supplement [5]. The niches of *L. ruminis* are variable, including human gastrointestinal tract [6], bovine rumen [4], piglet caecum and rectum [7], chicken digestive tract [8], sheep rumen [9], horse intestine [10], and dog intestine [11].

Many comparative genomic studies on *Lactobacillus* species have been reported, providing important information on their functional roles, metabolic capabilities, and evolutionary changes associated with niche adaptation [12]. The current development and application of genomic technologies has contributed to understanding the ecosystems of *L. ruminis* [13]. Carbohydrate is the main source of metabolic energy for lactobacilli, and is important for ecological adaptation [14]. Previous study described the carbohydrate utilization characteristics of nine *L. ruminis* stains, confirming the *L. ruminis* strains can express different carbohydrate utilization patterns [5].

Bacteriocin is an antibacterial peptide synthesized by bacterial ribosomes that provides its bacterial producer a competitive edge in its niche environment [15]. *L. ruminis* ATCC 27782 contains a gene cluster encoding a class IIa bacteriocin that has been shown to have antibacterial activity in vitro [16]. The identification of bacteriocin in vitro and in vivo is a time consuming and cumbersome procedure, whereas the in silico exploration of genes encoding bacteriocin biosynthesis provides a rapid and effective approach to identify putative bacteriocins.

Clustered regularly interspaced short palindromic repeats (CRISPR), which are highly variable sites, and found only in 40% bacteria [17], can provide acquired immunity resist the foreign genetic factors [18]. This CRISPR-based immunity allows bacteria to counter phage infections. In addition, it was reported that there is a relationship between the numbers of spacers in a CRISPR locus and phage resistance phenotype [19]. Previous research only identified CRISPR system existing in *L. ruminis* ATCC 27782 and ATCC 25644 without further analysis [17].

All the previous studies were carried out on animal-derived strains, however, to better understand the diversity, more strains from different origins should be include. The aim of the current work was to carry out comparative genomics analyses for *L. ruminis*, with more strains isolated from different niches, to explore the genetic diversity and potential host adaptation of the species.

## 2. Materials and Methods

### 2.1. Strains Ethics Approval Statement, Culturing, Genome Sequencing, and Data Assembly

This study was approved by the Ethics Committee in Jiangnan University, China (SYXK 2012-0002). All the fecal samples from healthy persons were for public health purposes and these were the only human materials used in present study. Written informed consent for the use of their fecal samples was obtained from the participants or their legal guardians. All of them conducted health questionnaires before sampling and no human experiments were involved. The collection of fecal samples had no risk of predictable harm or discomfort to the participants, and sampling of homemade fermented food and domestic animals were all consented by owners.

Eighty one strains of *L. ruminis*, previously isolated from animal and human feces from different regions, were cultured in De Man, Rogosa and Sharpe (MRS) medium [20] and incubated in a anaerobic workstation for 24 h. The draft genomes of all the strains were sequenced using llumina Hiseq × 10 platform (Illumina, San Diego, CA, USA) with a coverage depth no less than the genome 100 ×. The reads were assembled by SOAPde-novo [21]. Ten publicly available genomes listed in Table S1 from National Centre for Biotechnology Information (NCBI) (https://www.ncbi.nlm.nih.gov/) were used for comparison.

### 2.2. Genome Features Prediction and Annotation

The coding sequence (CDS) in the genome was predicted using Glimmer (http://ccb.jhu.edu/software/glimmer/index.shtml) [22]. The transfer RNA (tRNA) contained in the genome was predicted using tRNAscan-SE v2.0 software (http://trna.ucsc.edu/software/) [23]. The amino acid sequences were annotated using the Swiss-Prot [24] and RefSeq non-redundant proteins (NR) databases [25] by Diamond software, and the *E*-value was 1e-5.

### 2.3. Pan-Genome and Core-Genome Analysis

PGAP v1.2.1 was used to calculate the pan-genome and core-genome [26]. The protein sequences extracted from those 91 strains were aligned using Orthomcl software to make a Venn diagram (maintaining 50% identity; the cutoff *E*-value was 1e-4) [27]. 

### 2.4. Phylogenetic Comparison

All orthologous protein sequences of the 91 strains were extracted using Orthomcl-v2.0.9 software for clustering orthologous genes [28]. A phylogenetic tree was then constructed using the Neighbor-Joining (NJ) algorithm with default parameters in MEGA 7.0 software on MAFFT-aligned sequences [29], and decorated with Evolgenius (http://www.evolgenius.info/evolview/) [30].

### 2.5. Average Nucleotide Identity (ANI) Values

The ANI between any two genomes was calculated using a python script [31] (https://github.com/widdowquinn/pyani) and the resulting matrices were clustered and visualized using R packet heat map software (Kolde, R., Tartu, Estonia) [32].

### 2.6. Genotype/Phenotype Association Applied to Carbohydrate Metabolism 

The enzymes and genes involved in carbohydrate metabolism were annotated using the Carbohydrate Active Enzyme Database (CAZy, http://www.cazy.org/) [33], and the strains were visually clustered using HemI software [34].

Twenty-seven different sugars were selected for carbohydrate utilization analysis of *L. ruminis*. A stock solution of the carbohydrate was filtered (0.22 μm) into a carbohydrate-free MRS (cfMRS), and bromocresol purple was added to give a final fermentation concentration of 1%. A total of 1% of *L. ruminis* was inoculated into the medium, and the strain was activated twice to ensure its activity. After anaerobic incubation for 24 h at 37 °C, the utilization was judged by observing the color. The test was performed three times in duplicate on different occasions.

### 2.7. Bacteriocin Prediction

BAGEL4 is an online database that help mine and visualize ribosome-synthesized and post-translationally modified peptides and bacteriocin-producing gene clusters in the prokaryotic genome (http://bagel4.molgenrug.nl/index.php) [35]. On the BAGEL4 web server, a DNA nucleotide sequence was used as an input file. The conservation of RNA secondary structure was predicted by Weblogo (https://weblogo.berkeley.edu/logo.cgi) [36].

### 2.8. CRISPR Identification

CRISPRFinder (https://crisprcas.i2bc.paris-saclay.fr/CrisprCasFinder/Index) was used to discover CRISPR loci in *L. ruminis* genomes and predict CRISPR repeats and spacers [37]. Secondary structure prediction of repeat was performed by RNAfold (http://rna.tbi.univie.ac.at/cgi-bin/RNAWebSuite/RNAfold.cgi) [38]. According to the amino acid sequence of cas1 protein and the nucleotide sequence of CRISPR repeat sequence, phylogenetic analysis was carried out. The phylogenetic tree was constructed with 1000 repetitions using the p-distance model algorithm in the Neighbor-Joining Method in Molecular Evolutionary Genetics Analysis (MEGA) 7.0 software (Sudhir Kumar, Philadelphia, PA, USA). Number distribution of spacer sequences was plotted using GraphPad Prism 6 software (GraphPad Software Inc., San Diego, CA, USA).

### 2.9. Prophage Identification

PHASTER was used to identify the presence and composition integrity of prophages (http://phaster.ca/) [39]. The relevance between the number of spacer sequences and prophages was plotted using GraphPad Prism 6 software.

## 3. Results

### 3.1. Genomic Characterization of L. ruminis

In the previous work in our lab, 81 strains of *L. ruminis* strains were isolated from human feces (75 strains), piglets (three strains), dogs (two strains), and cows (one strain), which were sampled from fifteen cities in China (Table S1). Following whole genome sequencing, the draft genomes of these 81 strains, plus ten publicly available *L. ruminis* genomes (ATCC 27782 [40], ATCC 25644 [40], DSM 20403 [41], DPC 6830 [1], DPC 6832 [1], SPM0211 [42], S23 [1], bz_0080 [43], ICIS-540 and TF10-9AT) from NCBI GenBank Database, were analyzed. Those 91 *L. ruminis* strains have an average genome size of 2.16 Mb, 2310 genes and a GC content of 43.65 %, while the genome size of *L. ruminis* ranged between 1.94 Mb (S23) and 2.4 Mb (ICIS-540), in which the animal-derived strains exhibited smaller genome than that isolates from human. Similar results were found for gene number, in which gene number of animal-derived strains was relatively lower than that in human-derived strains. There was no obvious relationship between GC content and source host.

### 3.2. Pan-Genome and Core-Genome of L. ruminis

**Figure 1:**
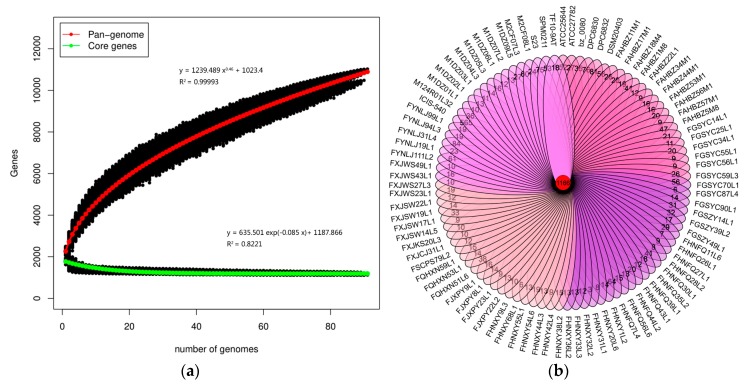
Pan-genome and core-genome of *L. ruminis*. (**a**) The pan-genome represented by the accumulated number of new genes against the number of genomes added. The core-genome represented by the accumulated number of genes attributed to the core-genomes against the number of added genomes. The mathematical functions of the pan- and core-genome based on 91 strains of *L. ruminis* are also shown on the graph; (**b**) Venn diagram of homologous genes of *L. ruminis*.

A pan-genome analysis was performed to determine the total number of different genes which were present in the *L. ruminis* genomes and the pan-genome curve displayed an asymptotic trend. The number of new gene increase gradually decreased from 466 at the beginning to 50 at the last group (Figure 1a). The mathematical function of the pan-genome displayed above the graph shows an exponential value less than 0.5, showing that the pan-genome was in a closing state. With the equation and genome number involved, the core genome of *L. ruminis* harbors 1188 genes (Figure 1a). The Venn diagram represented the specific and homologous core genes among all the 91 *L. ruminis* strains, showing the shared genes among all the strains assayed were 1166 genes, while the unique gene for each *L. ruminis* strain ranged from 3 to 565 genes (Figure 1b). 

### 3.3. Phylogenetic Analyses of L. ruminis

**Figure 2:**
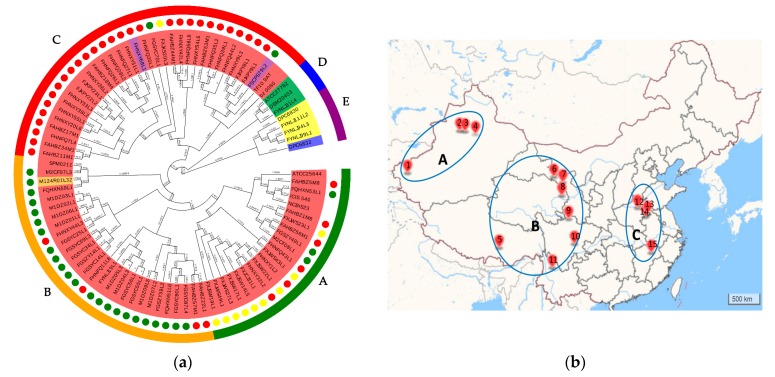
Phylogenetic analyses of *L. ruminis*. (**a**) The phylogenetic tree of *L. ruminis* based on orthologous genes. Unrooted phylogenies of the *L. ruminis* genomes were based on the multiple sequence alignment of core proteins and constructed with the neighbor-joining tree-building algorithm. Phylogenetic groups were highlighted in different colors. The text background color represented the source. Blue: horse; yellow: piglet; green: cow; red: human; purple: dog; orange: milk. Yellow circle: A region; green circle: B region; red circle: C region. The outer circle was divided into clade A–E, which was present with different colors and letters. (**b**) Source distribution map of isolating strains. According to the distance of strain source more than 1000 km, it can be divided into three regions. Each sampling point was labelled with different number. 1: Kashi; 2: Wusu; 3: Shawan; 4: Changji; 5: Dazi; 6: Zhangye; 7: Yongchang; 8: Xining; 9: Ruo’ergai; 10: Pengshan; 11: Lijiang; 12: Fengqiu; 13: Xiayi; 14: Bozhou; 15: Poyang.

To analyze the phylogenetic relationship of *L. ruminis* strains, a phylogenetic tree was created based on orthologue genes of 91 genomes that constituted to the core genome (Figure 2a). The resulting phylogenetic tree divided those strains in five clades (clades A to E). Clade E consisted of five strains, among which one strain was isolated from horses (DPC 6832) and another four strains were from piglets (DPC 6830, FYNLJ94L3, FYNLJ99L1, and FYNLJ111L2). Clade D included three strains, which were all bovine-derived strains. While the clade A to clade C gathered 83 *L. ruminis* strains, among which two isolates were from dogs, one isolate were from milk and the remained were from human feces. All those strains from different cities were divided into three regions based on the relative distance more than 1000 km of the source (Figure 2b). The strains in region A were mainly concentrated on clade A, and most of the strains in region B were clustered on clade B, while most strains in clade C were isolated from region C. 

### 3.4. ANI Values of L. ruminis

**Figure 3:**
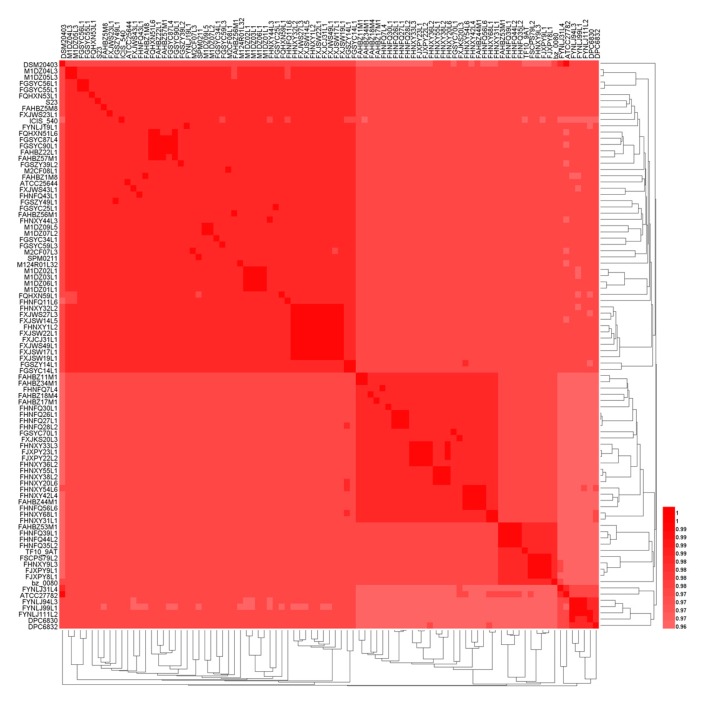
A heatmap based on average nucleotide identity (ANI) value of *L.ruminis.*

The average nucleotide identity (ANI) is a classical method for analyzing the unique species or potential subspecies existing among the strains within the same species. ANI values of those 91 genomes were carried out through pairwise comparison at the 95% threshold to further identify their species [44]. The results showed that all the 91 strains belonged to *L. ruminis*, and there was no potential subspecies for *L. ruminis* (Figure 3). The ANI values of *L. ruminis* strains from different sources were lower than that of strains from the same source.

### 3.5. Genotype/Phenotype Association Analysis for Carbohydrates Utilization in L. ruminis

**Figure 4:**
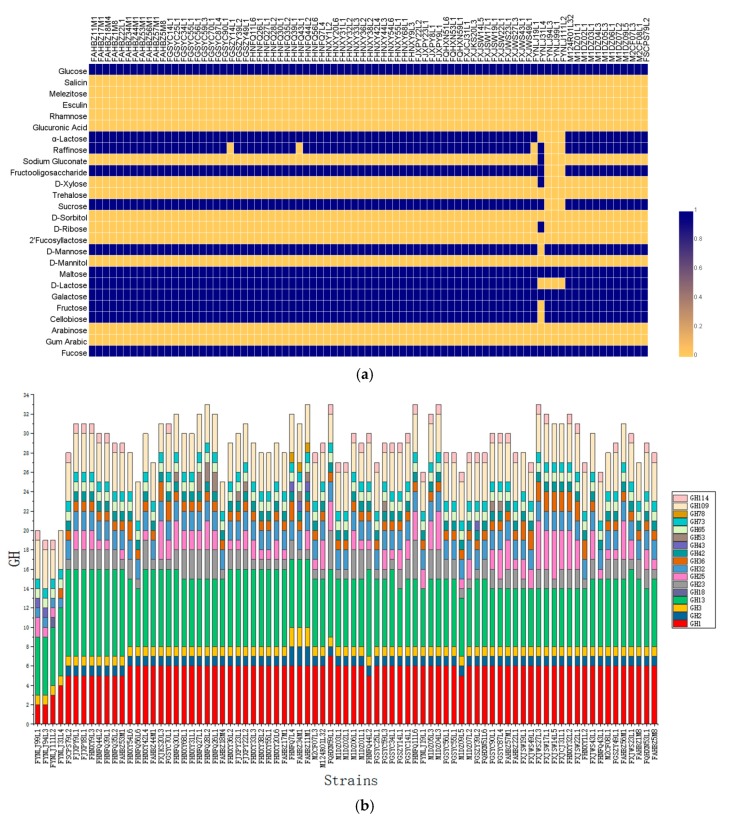
The fermentation profiles of *L. ruminis*. (**a**) Fermentation ability was indicated in blue for positive, while yellow for negative; (**b**) predicted glycosyl hydrolase (GH)-encoding gene content of *L. ruminis*.

Utilization profiles for all the 81 *L. ruminis* strains on 26 carbohydrates were performed (Figure 4a). Most strains (mainly human-derived) were able to ferment fucose, fructose, cellobiose, D-galactose, glucose, D-lactose, α-lactose, maltose, mannose, sucrose, raffinose, and fructooligosaccharide (FOS). However, none *L. ruminis* strains could utilize gum arabic, arabinose, D-mannitol, 2′-fucosyllactose (2’-FL), D-sorbitol, trehalose, glucuronic acid, rhamnose, esculin, melezitose, and salicin. The majority of animal-derived strains could poorly utilize lactose, sucrose, raffinose, and FOS, except for FYNLJ31L4. Among the 81 strains, only FYNLJ31L4 exhibited the ability to utilize D-ribose, D-xylose and sodium gluconate, while it cannot take advantage of other sugars such as cellobiose, D fructose, and D mannose that are utilized by remaining 80 *L. ruminis* strains.

The computational prediction of glycosyl hydrolases (GHs) in each genome was carried out using CAZy database to evaluate the carbohydrate fermentation genotype of *L. ruminis* (Figure 4b). This analysis identified seventeen GH families, highlighting the predominance of genes encoding GHs belonging to GH1, GH13, and GH109 families, which was in charge of the synthesis of α-glucosidase, β-glucosidase, α-N-acetylgalactosaminidase. The porcine-derived and bovine-derived strains did not consist of the GH2 and GH42 families, indicating that they could not synthesize β-galactosidase. In addition, the human-derived strains were shown to encode a higher number of GH families than all the animal-derived strains (Figure 4b). 

**Figure 5:**
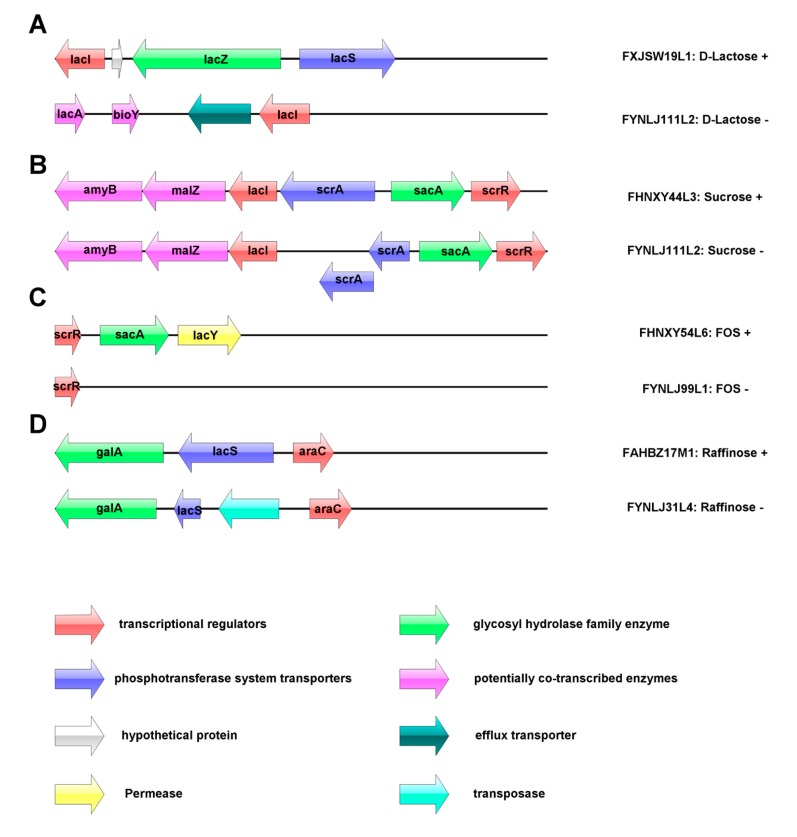
The carbohydrate utilization operon in *L. ruminis*. (**A**) lactose; (**B**) sucrose; (**C**) fructooligosaccharide (FOS); (**D**): raffinose.

Genotype and phenotype association analysis for utilization of carbohydrates was performed. Four putative carbohydrate utilization operons were annotated in the *L. ruminis* (Figure 5). The utilization of lactose was related to the β-galactosidase encoded by the GH2 and GH42 families, and the activity of the enzyme was predicted to be encoded by *lacZ* gene. The lactose operon of the porcine-derived and bovine-derived strains did not encode the *lacZ* gene, consistent with absence of GH2 and GH42 families in those strains, which led to led to their inability to utilize lactose (Figure 5A). The sucrose operon was predicted to be in charge of the transport of sucrose and hydrolysis of sucrose-6-phosphate, and in *L. ruminis* it appeared to relate to the β-fructofuranosidase and sucrose-6-phosphate hydrolase that belonged to the GH32 family, and those two enzymes were encoded by *sacA* in the sucrose operon. The majority of *L. ruminis* contained a complete sucrose operon, such as FHNXY44L3, but the porcine-derived strain, FYNLJ111L2, which could not utilize sucrose, was predicted due to the overlap and surplus of phosphotransferase system (PTS) transporter genes (*scrA*), resulting in termination of transcription (Figure 5B). β-fructofuranosidase has been identified as the key enzyme involved in FOS utilization in other *Lactobacillus*. The operon of FOS composed of *LacI* family transcriptional regulator, beta-fructofuranosidase and major facilitator super-family (MFS) transporter. The porcine-derived strains cannot use the FOS due to the deficiency of *sacA* genes which encoded β-fructofuranosidase (Figure 5C). Additionally, the utilization of raffinose was mainly regulated by active transport system (permease) and α-galactosidase. Insertion of the transposase in the raffinose operon of *L. ruminis* may affect its normal transcription leading to the inability to utilize raffinose (Figure 5D).

### 3.6. Prediction of Bacteriocin Operons in L. ruminis

BAGEL was used to identify the potential bacteriocin operon in those *L. ruminis* strains. In the study, 51 of 91 strains of *L. ruminis* produced bacteriocin (Table S2). Analysis performed on putative bacteriocin indicated that Class II bacteriocin was the majority, presenting in 47 *L. ruminis* strains, followed by Class I bacteriocin which was exist in thirteen strains and Class III bacteriocin was absent in all those 91 genomes. *L. ruminis* could synthesize five different bacteriocins, such as sactipeptides, plantaricin 423, leucocin, coagulin A and Hiracin JM79, which were belonged to class I and class II, respectively. Most of the strains with sactipeptides operon were located in cluster D in the phylogenetic tree, while the strains with Plantaricin 423 encoding genes were in cluster C. Additionally, most of the animal-derived strains in clusters A and B on the tree had the genes encoding coagulin A (Table S2).

**Figure 6:**
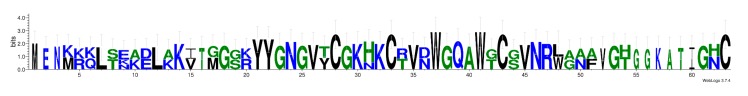
Relative frequency of conserved sequences from classII bacteriocin in *L. ruminis*.

**Figure 7:**
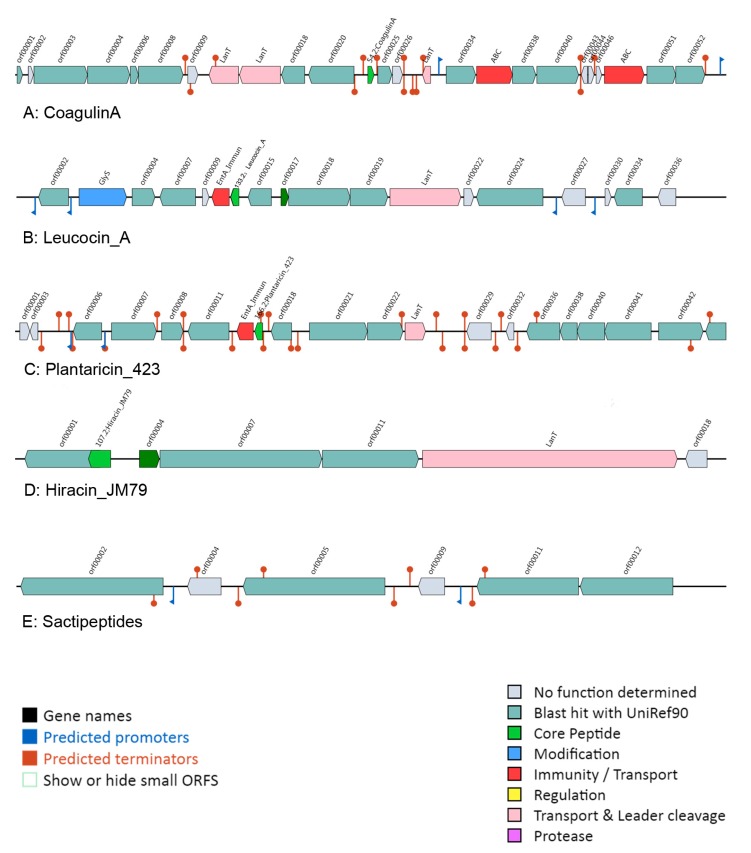
Bacteriocin gene operon in *L. ruminis.*

Four bacteriocins of class II showed high sequence conservation, and the precursor peptides included a conservative functional domain (YGNGVXCXXXXCXVXWXXA), that was classified to the class IIa bacteriocin (Figure 6). The biosynthetic gene clusters for class IIa bacteriocin were analyzed in *L. ruminis* (Figure 7). In addition, the gene cluster of class IIa bacteriocin generally contained three types of genes, including a structural prebacteriocin gene encoding core peptide, an immunity gene encoding an immunity protein and transporter genes. When BAGEL was used to analyze sactipeptides, the gene cluster only contained a structural gene encoding a putative peptide of cysteine residues that was connected with the synthesis of sactipeptides. However, the specific ATP-binding cassette (ABC) transporters were never present in the gene cluster of sactipeptides (Figure 7E). Therefore, the gene cluster encoding sactipeptides was incomplete and has no potential bacteriocin. 

### 3.7. Prediction of CRISPR/Cas Systems in L. ruminis

The CRISPR/Cas system was investigated in 91 genomes of *L. ruminis* strains by CRISPRFinder, and totally 59 CRISPR loci were identified in 49 out of 91 *L. ruminis* genomes (55%) (Table S3). Under some certain circumstances, there was no adjacent *cas* gene in the CRISPR region detected by CRISPR Finder, and these regions that were considered to be invalid were not further involved in the subsequent genetic analysis. Of the 49 genomes containing CRISPR, ten of them consisted of more than one CRISPR locus. Regarding the CRISPR/Cas system, subtypes I-B, I-E, I-C, IIA and IIIA were identified, in which Type II was the most abundant and with IIA being the most dominant subtype. Only *L. ruminis* DPC6832 contained a subtype I-B CRISPR/Cas system.

**Figure 8:**
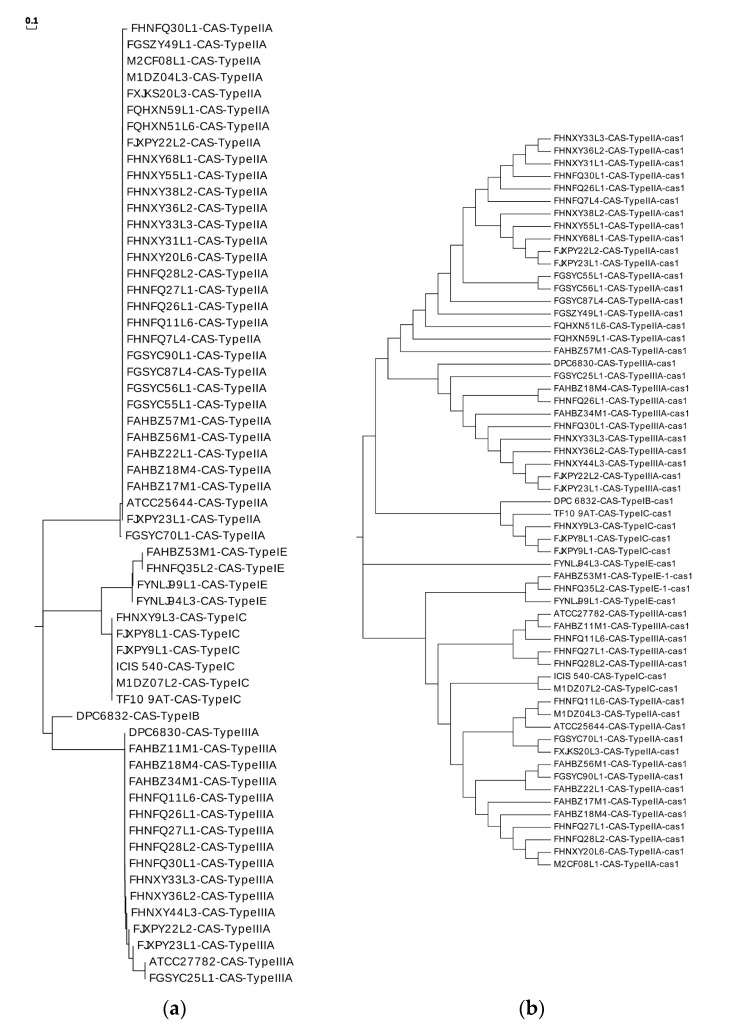
Phylogenetic analysis of clustered regularly interspaced short palindromic repeats /CRISPR-associated (CRISPR/Cas) system. (**a**) Phylogenetic tree of direct repeats in *L. ruminis*; (**b**) the CRISPR/Cas classification onto the phylogenetic tree of *Cas1*.

**Figure 9:**
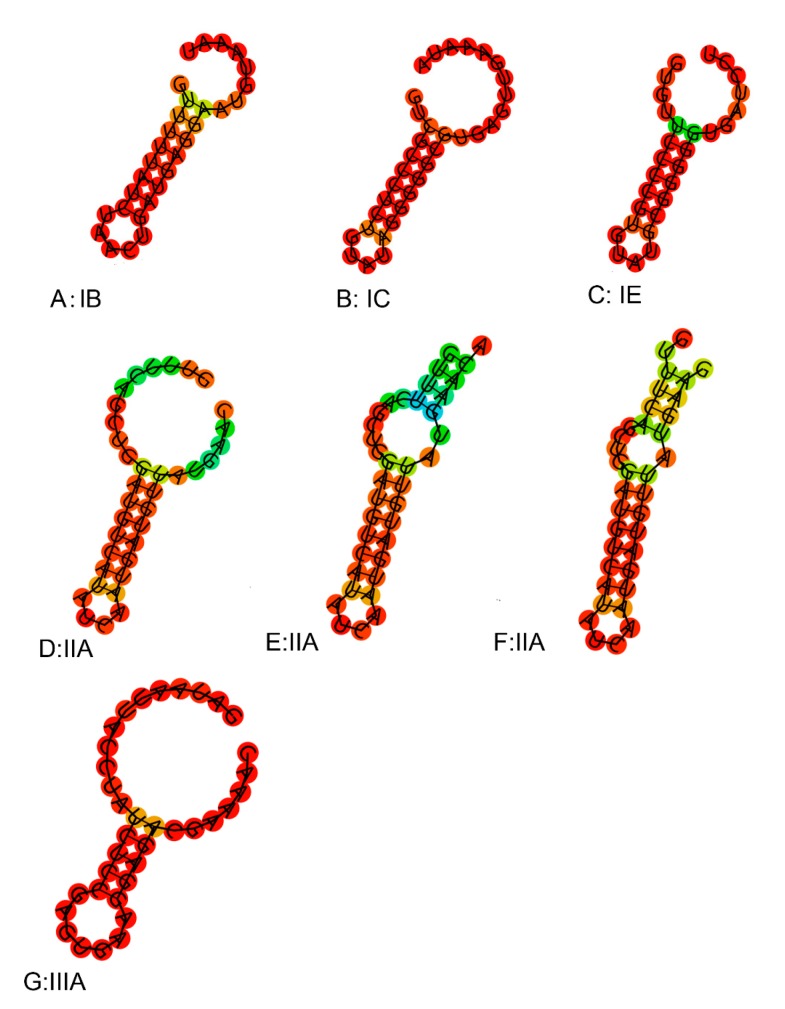
Secondary structure of repeats in *L. ruminis.*

The number of spacer sequences in the CRISPR loci of different subtypes was analyzed (Figure.S1), whose number in the subtype IIA locus varied greatly, up to 66, at least five, with an average of about 24. The number of subtype IC spacer sequences was the lowest. Repeat sequences of the same type of CRISPR/Cas system showed high homology in the phylogenetic tree (Figure 8a). By predicting its secondary structure, repeat sequences can be better explored (Figure 9). The difference between the repeats of the types IB, IC, IE, and IIIA identified in *L. ruminis* were only a few base pair, with mainly the same frequency and secondary structure. Therefore, only one secondary structure was performed for the four types. Repeat sequences from the IIA subtype were more variable, with a total of three structures. When focusing on the RNA secondary structure of the repeat sequence of the CRISPR/Cas system in *L. ruminis* genomes, it was found that both ends of the repeat sequence contained a large loop and a small loop (Figure 9D–F), which was a typical stem-loop stable structure. Among these subtypes, IE and IIA contain G:U base pairs which were classical of conserved RNA secondary structures (Figure 9C–F). CRISPR/Cas loci onto the Cas1 tree demonstrated a considerable agreement between the phylogeny of Cas1 and locus types and subtypes (Figure 8b). *Cas1* gene of subtype I-E was strictly monophyletic, while *cas1* gene of subtype I-C, II-A and III-A was largely monophyletic, with a few exceptions. 

### 3.8. Prediction of Prophage in L. ruminis

**Figure 10:**
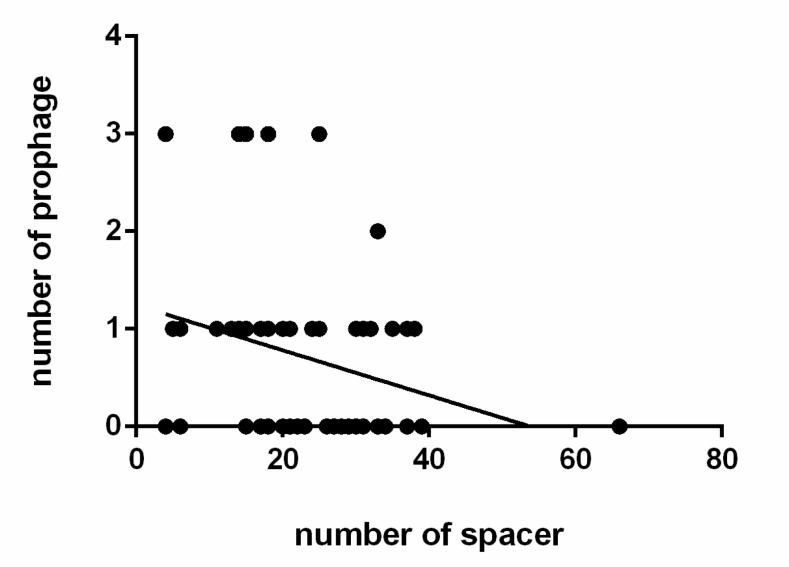
Correlation between the number of spacer and prophage in *L. ruminis*. Mapping the number of spacers in each strain to the corresponding amount of prophage (*p* = 0.0241).

The prophages in the *L. ruminis* stains were predicted by PHASTER, and the results were listed in Table S4. Those prophages were predicted to be ‘intact’, ‘incomplete’ or ‘questionable’. Incomplete and questionable described the CDSs associated with the prophage gene cluster, but they did not correctly define prophage. Fifty-five intact prophages were identified in 40 out of 91 *L. ruminis* genomes (44%) (Table S4). Among them, 28 *L. ruminis* strains carried only one intact prophage, nine strains carried two prophages, and three strains carried three prophages. Interestingly, some prophage gene clusters which encoding structural and lysis components were identified as questionable or incomplete. In addition, by researching the correlation between the number of spacer sequences and the prophage, it was found that the relationship was negatively correlated in *L. ruminis* strains (Figure 10).

## 4. Discussion

*L. ruminis* is an important member of the intestinal tract of mammals and is also considered as a native species of the human intestine. In this study, the genomic variety and niche adaptability of *L. ruminis* were explored. *L. ruminis* showed a lower genome size than other *Lactobacillus* that belonged to free living and nomadic life style, such as *Lactobacillus rhamnosus* [45], *Lactobacillus casei* [46], and *Lactobacillus buchneri* [47], which corresponded to the characteristics of the host-adapted lifestyle. According to the genetic characteristics of those 91 *L. ruminis* strains, both genome size and genes number among those strains isolated from different sources differed, which was similar to that in *L. salivarius*. Genome size and genes number of piglet-derived *L. salivarius* strains were larger than those of the human-derived and chicken-derived strains, while the GC content was not different between isolates in relation to their hosts [48]. And the pan-genome and core-genome analysis showed a closed pan-genome for *L. ruminis* species, pointing out that an enough number of strains had been included in the current research to sufficiently describe the genetic diversity in *L. ruminis* species.

In the previous evolutionary analysis of *Lactobacillus* genus, some species separated in phylogenetic clusters that were highly reflective of host source, such as *L. reuteri* [49], *L. ruminis* [1] and *Lactobacillus johnsonii* [50]. Similar results had been obtained in this study. The evolutionary relationship between porcine-derived and horse-derived *L. ruminis* was relatively close, probably because both of them were non-ruminants and from the same geographical niche. Remarkably, there was a closer phylogenetic relationship between the human-derived and bovine-derived strains, which indicated the strains isolated from them might have a common ancestor, although the host origin was divergent. Reasonably, human have the monogastric digestive system, which should be more similar to non-ruminants (piglet and horse) [51]. This phenomenon may indicate that the niche difference between ruminant and non-ruminant species did not limit the evolutionary potential of *L. ruminis*. It was worth noting that two dog-derived *L. ruminis* strains and human-derived *L. ruminis* were on the same clades, which may be due to the similar living environment with human and special dietary characteristics. Interestingly, the human-derived *L. ruminis* was distinguished by their geographical origin. *L. ruminis* from similar geographical locations has a more similar ecological environment, and we assumed that they represented ecological ecotypes. Ecotypes were populations suited to specific environmental conditions whose members were both genetically and ecologically similar [52]. Therefore, it was suspected that strains from the same geographical origin were more likely to cause genetic drift that promoted the genetic identity of ecological types [52].

*L. ruminis* was previously described as a homofermentative bacterium, with the ability to ferment cellobiose, galactose, maltose, mannose, raffinose, salicin, and sucrose [2]. In the current study, the 81 strains of *L. ruminis* were unable to utilise salicin as a carbon source. The *salCAB* operon that encoded two aryl-β-glucosidases [53], required for growth on salicin, was not exist in 81 *L. ruminis* genomes. Porcine-derived *L. ruminis* (DPC 6831) had also been reported with the capacity to ferment esculin [1]. However, we found no growth for any of the 81 *L. ruminis* strains with it as the sole carbon source. Similarly, utilization of esculin was also not found in the other nine strains (four bovine-derived strains and five human-derived strains) of *L. ruminis* [5]. Among the 81 strains, only bovine-derived strains (FYNLJ31L4) can ferment ribose. In the previously reported literature, only *L. ruminis* derived from fermented fish (NRIC 1689) can ferment ribose [54]. Therefore, it was hypothesized that the key enzyme transaldolase (EC 2.2.1.2) gene [55] encoding ribose metabolism might be lost in most *L. ruminis*. β-glucooligosaccharides such as cellobiose were generally transported and hydrolysed using the cellobiose PTS and β-glucosidase enzymes. *L. ruminis* is a species that is normally capable of fermenting prebiotic compounds including FOS and raffinose. However, piglet-derived strains did not have the ability to ferment raffinose [5]. *L. ruminis* from the same niche showed high consistency in sugar fermentation. The results reflected in the sugar fermentation analysis of porcine-derived and horse-derived *L. ruminis* [1]. Meanwhile, it was worth noting that the human-derived strains were shown to encode a higher number of GH families than the animal-derived strains. Accordingly, *L. ruminis* strains from human represented a broad variability in GH enzymes which would be corresponding to dietary diversity of human hosts. To some extent, these differences reflected not only the genomic variety of *L. ruminis*, but also niches adaptation through the acquisition or loss of metabolically related genes [56]. 

Bacteriocin is small antimicrobial peptide produced by many bacteria, including *Lactobacillus*, which may display either a narrow spectrum against closely related species or broad spectrum to species that belonged to different genera [57]. Heretofore, the well-known bacteriocin produced by *L. ruminis* belonged to class IIa bacteriocin, in which *L. ruminis* ATCC 27782 generated a Class II pediocin-like bacteriocin [17]. Sactipeptides is a sulphur-to-α-carbon-containing peptides, known as assactibiotics when it showed antibacterial activity [58]. Notably, the common characteristics in the sactibiotic gene clusters composed of the immunity proteins, structural genes, transporters, and S-adenosylmethionine enzymes including a classic conserved domain [59]. In addition, the precursor peptide containing one or more cysteine residues and the ABC-type bacteriocin transport system were considered to be potential biosynthetic gene clusters encoding sactipeptides [16]. In the current study, the sactipeptides of class I bacteriocin identified by BAGEL lacked a transporter and was not considered to be a credible bacteriocin gene operon. There had been no sactipeptide from a lactic acid bacteria strain characterized yet and only presumed bacteriocin clusters had been identified via in silico analysis [60], which needs further investigation.

This study researched the variety and distribution of CRISPR/Cas loci in 91 strains of *L. ruminis*, and the number of spacer sequences reflected the activity of the CRISPR system. An active CRISPR/Cas system has been shown to be able to continuously obtain spacer sequences. Conversely, the spacer sequence will be deleted in order to retain the activity of the CRISPR/Cas system in the absence of selection pressure [61]. From the number of spacer sequences, it can be inferred that the subtype IIA locus in *L. ruminis* was more active and had better ability to against the insertion of exogenous gene. The similar results were obtained in previous studies [62]. The presence of the G:U base pair highlighted the significance of stem loops in the repeat sequence for the function of CRISPRs [63]. The high denaturation of the CRISPR locus was illustrated by the variety observed in the number and sequence of the CRISPR spacers despite the CRISPR repeat conservation and Cas homology. In addition, it was interesting to note that incomplete and questionable prophages contained some phage components. For instance, there were some gene clusters that can encode structures, lysin, or lysis modules, whereas prophages were questionable or incomplete. It is inferred that retention of interfering prophage residues may be beneficial to the host. The discovery that bacteria containingincomplete prophages can have compatible functions such as bacteriocin supported this viewpoint [64]. *Streptococcus pyogenes* stains that lacked of CRISPR system contained evidently more prophages than CRISPR possessing strains [65]. And similar result was found in *L. ruminis* in the current work. The inverse proportion between number of spacers and phages of *L. ruminis* was obvious. It can be presumed that strains with a high amount of CRISPR/Cas system were more advantageous as compared with strains without these, when it comes to use DNA as nutrient.

## 5. Conclusions

In this study, the genome sequences of 91 *L. ruminis* strains provided a basis for functional gene analysis of this species. As a host adaptive lifestyle, the difference in niches had a greater impact on the evolution of bacterial genes. Adaptation to different host intestinal competition environments included the utilization of carbohydrates, the production of bacteriocin, and the presumed large number of CRISPR loci and prophage, which will contribute to the persistence of *L. ruminis* in the native colonization of the gastrointestinal tract.

## Supplementary Materials

The following are available online at https://www.mdpi.com/2073-4425/11/1/70/s1, Figure S1: Number distribution of spacer sequences of different subtypes in *L. ruminis*; Table S1: *L. ruminis* strains and genomes in this study; Table S2: Predicted bacteriocin in *L. ruminis*; Table S3: CRISPR repeat/spacer in *L. ruminis*; Table S4: Predicted prophage based on PHASTER.

## Abbreviations

ABC transporter	ATP-binding cassette transporter
ANI	Average Nucleotide Identity
CAZy	Carbohydrate Active enzymes
CDS	Coding sequence
cfMRS	Carbohydrate-free MRS
CRISPR	Clustered regularly interspaced short palindromic repeats
2′-FL	2′-Fucosyllactose
FOS	Fructooligosaccharide
GHs	Glycoside Hydrolases
*L. ruminis*	*Lactobacillus ruminis*
MFS	Major Facilitator Super-family
MRS	De Man, Rogosa and Sharpe
NCBI	National Center for Biotechnology Information
NR	RefSeq non-redundant proteins
PTS	Phosphotransferase System
tRNA	Transfer RNA
